# Clinical and Procedural Characteristics and In-Hospital Outcomes of Stent Thrombosis Following Primary Percutaneous Coronary Intervention (PCI)

**DOI:** 10.7759/cureus.104650

**Published:** 2026-03-04

**Authors:** Mudasir Habib, Muzdalfa Parvez, Kiflain Hassan, Inam Ullah, Muhammad Haroon, Syed Atta Ullah

**Affiliations:** 1 Department of Cardiology, Khyber Teaching Hospital, Peshawar, PAK; 2 Department of Cardiology, District Headquarters Hospital, Kohat Development Authority (KDA), Kohat, PAK; 3 Department of Cardiology, Hayatabad Medical Complex, Peshawar, PAK; 4 Department of Internal Medicine, Saidu Group of Teaching Hospitals, Swat, PAK; 5 Department of Internal Medicine, District Headquarters Hospital, Kohat Development Authority (KDA), Kohat, PAK; 6 Department of Medicine, Khyber Institute of Medical Sciences, Kohat, PAK

**Keywords:** acute stent thrombosis, coronary intervention, in-hospital outcomes, pakistan, primary pci, stemi

## Abstract

Introduction

Early stent thrombosis (ST) (≤30 days) following primary percutaneous coronary intervention (PCI) for ST-segment elevation myocardial infarction (STEMI) remains a rare but serious complication. Despite advances in stent technology and antithrombotic therapy, this condition continues to be associated with high morbidity and mortality, particularly in resource-limited settings where local data are scarce.

Purpose

The purpose of this study is to evaluate the clinical characteristics, procedural factors, and in-hospital outcomes of patients with early stent thrombosis and to compare these findings with matched controls without stent thrombosis in a tertiary care cardiac center in Peshawar, Pakistan.

Materials and methods

This retrospective observational study included adult patients undergoing primary PCI who developed acute stent thrombosis (AST) (≤24 hours) or subacute stent thrombosis (24 hours to 30 days) during the same hospitalization. Data were collected from medical records, catheterization laboratory reports, and electronic hospital systems using a structured questionnaire. SPSS version 27.0 (IBM Corp., Armonk, NY) was used for statistical analysis, with comparisons made between cases and matched controls.

Results

A total of 80 patients with early stent thrombosis and 160 matched controls were analyzed. The majority were men (62/80, 77.5%). Common baseline cardiovascular risk factors included diabetes mellitus (28/80, 35%), hypertension (42/80, 52.5%), dyslipidemia (34/80, 42.5%), smoking (26/80, 32.5%), and prior coronary artery disease (12/80, 15%). Acute stent thrombosis occurred predominantly within 24 hours post-PCI (16/80, 20%). Procedural characteristics, including stent type, vascular access, and adjunctive therapies, were largely similar between sexes. Repeat PCI was the primary management strategy (62/80, 77.5%), and while most patients survived to discharge, a subset experienced serious in-hospital complications: heart failure (18/80, 22.5%), recurrent ischemia (10/80, 12.5%), arrhythmias (12/80, 15%), acute kidney injury (8/80, 10%), or bleeding events (6/80, 7.5%). Procedural factors associated with early stent thrombosis included high thrombus burden, suboptimal stent expansion, overlapping stents, and reduced post-procedural thrombolysis in myocardial infarction (TIMI) flow.

Conclusion

Early stent thrombosis after primary PCI remains an uncommon but potentially life-threatening complication with substantial in-hospital morbidity. Timely recognition and prompt intervention, primarily through repeat PCI, are essential to improve outcomes. These findings highlight the need for larger, multicenter studies to identify modifiable patient- and procedure-related risk factors, particularly in local populations, to enhance prevention and management strategies for early stent thrombosis.

## Introduction

Stent thrombosis (ST) remains one of the most serious complications of primary percutaneous coronary intervention (PCI), particularly in patients presenting with ST-segment elevation myocardial infarction (STEMI). Despite advances in stent technology, dual antiplatelet therapy, and procedural techniques, ST continues to cause significant morbidity and mortality. Early ST, defined as occurring within 30 days of PCI (including acute and subacute events), is strongly associated with poor clinical outcomes.

Heestermans et al. reported that among 5,842 patients with STEMI undergoing primary PCI, 201 patients (3.5%) experienced definite early stent thrombosis, including 97 (1.7%) acute and 104 (1.8%) subacute events [1]. Procedural predictors of acute ST included stent undersizing, post-procedural dissection, and smaller stent diameters, while the lack of clopidogrel within the first 30 days was the strongest predictor for subacute ST. Ullah et al. analyzed 153 PCI patients and found that 20.9% of ST events were acute, 70.6% subacute, and 8.5% late, with significant predictors including diabetes mellitus, hypertension, acute coronary syndrome presentation, stent under-expansion, and non-adherence to dual antiplatelet therapy [2]. Cosansu et al. demonstrated that in 616 patients with STEMI with small vessel coronary artery disease, direct stenting reduced stent thrombosis rates from 4.2% with conventional stenting to 1.0% (p = 0.04) over two years [3]. Direct stenting also improved post-procedural thrombolysis in myocardial infarction (TIMI) 3 flow and reduced edge dissections, procedural time, radiation exposure, and contrast usage. Collectively, these data highlight that careful stent sizing, meticulous procedural technique, strict adherence to dual antiplatelet therapy, and the optimal management of comorbidities are critical to reducing early ST and improving both short- and long-term outcomes in patients with STEMI undergoing PCI.

Despite the increasing use of PCI as the standard of care for STEMI in tertiary cardiac centers, there is limited data in Pakistan regarding the clinical profile, angiographic characteristics, procedural factors, and in-hospital outcomes of early stent thrombosis following primary PCI. Available evidence suggests that high-risk patients with STEMI in resource-constrained settings remain particularly vulnerable to this complication, which is associated with increased mortality and impaired post-PCI coronary flow [4,5].

Previous retrospective studies in Pakistan have examined procedural aspects and risk factors related to stent thrombosis in post-PCI cohorts, highlighting the complexity of this condition and the need for systematic evaluation in local practice settings [6]. However, comprehensive data specifically focusing on early stent thrombosis (≤30 days) among patients undergoing primary PCI remain scarce. Given the high prevalence of coronary artery disease and the widespread use of PCI in tertiary care centers across the country, a detailed analysis of clinical presentation, angiographic findings, management strategies, and in-hospital outcomes is essential. Understanding these factors is crucial for identifying modifiable risk determinants, optimizing interventional strategies, and improving clinical outcomes. The primary aim of this study was to evaluate the clinical characteristics, procedural factors, and in-hospital outcomes of adult patients who developed early stent thrombosis (≤30 days) following primary PCI in a tertiary cardiac center in Peshawar, Pakistan.

## Materials and methods

Study design and definitions

This retrospective observational study was conducted at a tertiary care cardiac facility in Peshawar, Pakistan, from January to July 2025, using previously recorded data. The study included adult patients who underwent first-time PCI as first-line treatment for acute STEMI between January 2023 and December 2024. Stent thrombosis (ST) was identified during the same hospitalization and defined as angiographic or autopsy-confirmed thrombus formation within or adjacent to the stent, accompanied by clinical deterioration such as recurrent chest pain, electrocardiographic changes, or myocardial infarction (MI). In terms of timing, ST is classified as acute (≤24 hours post-PCI), subacute (24 hours to one month), early (≤1 month, encompassing both acute and subacute), late (1-12 months), and very late (>12 months) [7,8]. Only early ST cases (acute and subacute) were included in this study for analysis.

Inclusion and exclusion criteria

Patients were eligible for the study if they were adults who underwent primary PCI for STEMI and subsequently developed early stent thrombosis (≤1 month) during the same hospitalization.

Patients were excluded if they underwent elective or rescue PCI, developed late (1-12 months) or very late (>12 months) stent thrombosis, or had incomplete or inadequate procedural or clinical records. These criteria were applied to ensure a well-defined, homogeneous study population and accurate data collection for acute stent thrombosis (AST) following primary PCI. Missing or incomplete records were excluded from analysis.

Study sample

During the study period (January 2023 to December 2024), a total of 645 primary PCI procedures were performed, of which 80 patients (12.4%) developed early stent thrombosis. Acute ST (≤24 hours) occurred in 16 patients (2.5%), while subacute ST (24 hours to one month) was observed in 64 patients (9.9%). A control group of 160 PCI patients without stent thrombosis was selected at a 2:1 ratio for comparative analyses (Table 1).

**Table 1:** Incidence of Early Stent Thrombosis Early ST: stent thrombosis occurring within 30 days after PCI. Acute ST: ≤24 hours. Subacute ST: 24 hours to 30 days. Control group: matched PCI patients without ST ST, stent thrombosis; PCI, percutaneous coronary intervention

Parameter	Value
Total primary PCI procedures (January 2023 to December 2024)	645
Total early stent thrombosis cases	80
Overall early ST incidence	12.4%
Acute ST (≤24 hours)	16 (2.5%)
Subacute ST (24 hours to 30 days)	64 (9.9%)
Control group selected	160 (2:1 ratio)

Data collection instrument

A standardized questionnaire was designed specifically for this study and validated with interventional cardiologists to ensure applicability to local clinical practice (Table 2). The questionnaire was pilot-tested on a small sample of records to confirm clarity, consistency, and completeness, and modifications were made before full data collection. Retrospective data were obtained from hospital medical records, catheterization laboratory findings, nursing records, and electronic monitoring systems. Patient identifiers were removed to maintain confidentiality.

**Table 2:** Data Collection Instrument of Stent Thrombosis in Patients Undergoing Primary PCI PCI, percutaneous coronary intervention; CABG, coronary artery bypass grafting; LV, Left ventricular; LAD, left anterior descending; RCA, right coronary artery; LCX, left circumflex; CCU, coronary care unit; ECG, electrocardiogram; bpm, beats per minute

Section	Variable	Response/Unit
Patient identification and demographics	Study code number	__________
	Hospital registration number	__________
	Date of admission	__________
	Date of primary PCI	__________
	Time of primary PCI	__________
	Age	__________ years
	Sex: male	☐ Yes ☐ No
	Sex: female	☐ Yes ☐ No
	Weight	__________ kg
	Height	__________ cm
	Body mass index	__________ kg/m²
	Residence: urban	☐ Yes ☐ No
	Residence: rural	☐ Yes ☐ No
	Referral status: direct	☐ Yes ☐ No
	Referral status: referred	☐ Yes ☐ No
Baseline medical and cardiovascular history	Hypertension	☐ Yes ☐ No
	Duration of hypertension	__________ years
	Diabetes mellitus	☐ Yes ☐ No
	Diabetes treatment: oral	☐ Yes ☐ No
	Diabetes treatment: insulin	☐ Yes ☐ No
	Diabetes treatment: both	☐ Yes ☐ No
	Smoking: current	☐ Yes ☐ No
	Smoking: former	☐ Yes ☐ No
	Smoking: never	☐ Yes ☐ No
	Smokeless tobacco use	☐ Yes ☐ No
	Dyslipidemia	☐ Yes ☐ No
	Chronic kidney disease	☐ Yes ☐ No
	Previous myocardial infarction	☐ Yes ☐ No
	Previous PCI	☐ Yes ☐ No
	Previous CABG	☐ Yes ☐ No
Pre-hospital medication and compliance	Pre-hospital antiplatelet use	☐ Yes ☐ No
	Antiplatelet agent type	__________
	Anticoagulant use	☐ Yes ☐ No
	Medication non-adherence	☐ Yes ☐ No
	Recent drug interruption	☐ Yes ☐ No
Clinical presentation and admission	Time of symptom onset	__________
	Duration of chest pain	__________ hours
	Chest pain: typical	☐ Yes ☐ No
	Chest pain: atypical	☐ Yes ☐ No
	Dyspnea	☐ Yes ☐ No
	Syncope	☐ Yes ☐ No
	Nausea/vomiting	☐ Yes ☐ No
	Transport: ambulance	☐ Yes ☐ No
	Transport: private	☐ Yes ☐ No
	Door-to-procedure time	__________ minutes
	Heart rate	__________ bpm
	Systolic blood pressure	__________ mmHg
	Diastolic blood pressure	__________ mmHg
	Oxygen saturation	__________ %
	Signs of heart failure	☐ Yes ☐ No
	Cardiogenic shock	☐ Yes ☐ No
Electrocardiographic and laboratory findings	Infarct location: anterior	☐ Yes ☐ No
	Infarct location: inferior	☐ Yes ☐ No
	Infarct location: lateral	☐ Yes ☐ No
	Infarct location: posterior	☐ Yes ☐ No
	Arrhythmia on admission ECG	☐ Yes ☐ No
	Initial cardiac enzymes	__________
	Serum creatinine	__________ mg/dL
	Blood urea	__________ mg/dL
	Hemoglobin	__________ g/dL
	Platelet count	__________ ×10⁹/L
	Random blood glucose	__________ mg/dL
Echocardiographic findings	LV systolic function: normal	☐ Yes ☐ No
	LV systolic function: mild	☐ Yes ☐ No
	LV systolic function: moderate	☐ Yes ☐ No
	LV systolic function: severe	☐ Yes ☐ No
	Regional wall motion abnormality	☐ Yes ☐ No
	Mechanical complications	☐ Yes ☐ No
Angiographic characteristics	Infarct-related artery: LAD	☐ Yes ☐ No
	Infarct-related artery: RCA	☐ Yes ☐ No
	Infarct-related artery: LCX	☐ Yes ☐ No
	Diseased vessels: one	☐ Yes ☐ No
	Diseased vessels: two	☐ Yes ☐ No
	Diseased vessels: three	☐ Yes ☐ No
	Lesion location: proximal	☐ Yes ☐ No
	Lesion location: mid	☐ Yes ☐ No
	Lesion location: distal	☐ Yes ☐ No
	Thrombus burden: low	☐ Yes ☐ No
	Thrombus burden: moderate	☐ Yes ☐ No
	Thrombus burden: high	☐ Yes ☐ No
	Collateral flow present	☐ Yes ☐ No
Procedural characteristics	Vascular access: radial	☐ Yes ☐ No
	Vascular access: femoral	☐ Yes ☐ No
	Pre-dilatation performed	☐ Yes ☐ No
	Number of stents deployed	__________
	Stent type: drug-eluting	☐ Yes ☐ No
	Stent type: bare metal	☐ Yes ☐ No
	Stent length	__________ mm
	Stent diameter	__________ mm
	Post-dilatation performed	☐ Yes ☐ No
Adjunctive therapies	Thrombus aspiration used	☐ Yes ☐ No
	Intracoronary medications used	☐ Yes ☐ No
	Anticoagulant administered	__________
	Additional antiplatelet agent used	☐ Yes ☐ No
Acute stent thrombosis details	Time of thrombosis detection	__________
	Clinical chest pain	☐ Yes ☐ No
	ECG changes	☐ Yes ☐ No
	Hemodynamic instability	☐ Yes ☐ No
	Angiographic confirmation	☐ Yes ☐ No
	Immediate management strategy	__________
In-hospital complications and re-interventions	Recurrent ischemia	☐ Yes ☐ No
	Heart failure	☐ Yes ☐ No
	Arrhythmias	☐ Yes ☐ No
	Bleeding events	☐ Yes ☐ No
	Acute kidney injury	☐ Yes ☐ No
	Repeat coronary intervention	☐ Yes ☐ No
Hospital course and discharge outcomes	CCU stay	__________ days
	Total hospital stay	__________ days
	Mechanical support required	☐ Yes ☐ No
	Survival at discharge: alive	☐ Yes ☐ No
	Survival at discharge: deceased	☐ Yes ☐ No
	Discharge medications prescribed	__________

The questionnaire included multiple domains: study code and hospital registration number, date and time of PCI, demographic details (age, sex, weight, height, body mass index {BMI}, place of residence, and referral status), baseline medical and cardiovascular history (hypertension, diabetes mellitus and treatment, dyslipidemia, chronic kidney disease, past MI, previous PCI or coronary artery bypass grafting {CABG}, long-term medications, and non-adherence to antiplatelet therapy), pre-hospital antiplatelet and anticoagulant treatment, recent medication interruptions, and compliance history.

Clinical presentation on admission included the time of symptom onset, the duration and type of chest pain, accompanying symptoms, transport method, and door-to-procedure time. Vital signs, consciousness, evidence of pulmonary edema or heart failure, and need for inotropic or mechanical support were recorded. Electrocardiographic (ECG) findings and infarct site were also documented.

Laboratory parameters included initial cardiac biomarkers, serum creatinine, blood urea, hemoglobin, platelet count, and random blood glucose. Echocardiographic evaluation captured left ventricular systolic function, regional wall motion abnormalities, and mechanical complications. Procedural and angiographic characteristics included infarct-related artery, the number of diseased vessels, lesion location, thrombus burden, the number and type of stents, stent length and diameter, pre- and post-dilatation, adjunctive intracoronary medications, and antiplatelet and anticoagulant therapy administered before, during, and after the procedure.

In-hospital outcomes included recurrent ischemia, ECG changes, repeat coronary intervention, heart failure, arrhythmias, bleeding events, acute kidney injury, ICU and total hospital stay, need for mechanical support, survival at discharge, and discharge medications.

Follow-up and bias minimization

Patients were followed throughout hospitalization until discharge or in-hospital death. All eligible patients were recruited consecutively to minimize selection bias. Data were cross-checked using multiple sources (medical charts, catheterization records, and electronic records) to reduce information bias. Data extraction was performed by trained personnel, and all entries were reviewed by a senior cardiologist to ensure accuracy and reduce observer bias.

Data management and statistical analysis

All data were entered into a secure database and analyzed using SPSS version 27 (IBM Corp., Armonk, NY). Continuous variables were summarized as mean ± SD or median with interquartile range (IQR), depending on distribution, while categorical variables were expressed as frequencies and percentages. Comparisons between cases (early ST) and controls (PCI patients without ST) were performed using the independent t-test or Mann-Whitney U test for continuous variables, and chi-square (χ²) or Fisher’s exact test for categorical variables. Odds ratios (ORs) with 95% confidence intervals (CIs) were calculated to assess the strength of the association between clinical, procedural, and demographic variables and early ST occurrence. Statistical significance was set at p < 0.05.

Ethical considerations

Informed consent for the treatment and use of anonymized data for research and publication was obtained. The study was approved by the Ethical Review Board of Hayatabad Medical Complex, Peshawar (approval number: 857/CD/HMC/2024). The Ethical Review Board reviewed the study in accordance with the Declaration of Helsinki (2013) and confirmed that it met all ethical requirements.

## Results

The study included 80 patients who developed early stent thrombosis (cases) and 160 matched controls. The mean age was similar between cases and controls (58.0 ± 10.1 versus 57.5 ± 9.8 years; p = 0.72), and the proportion of men was comparable (62/80, 77.5%, versus 125/160, 78.1%; p = 0.92). No significant differences were observed for BMI, hypertension (42/80, 52.5%, versus 75/160, 46.9%; p = 0.38), dyslipidemia (34/80, 42.5%, versus 58/160, 36.3%; p = 0.34), prior MI (12/80, 15.0%, versus 18/160, 11.3%; p = 0.41), or Killip classes III-IV at presentation (15/80, 18.8%, versus 18/160, 11.3%; p = 0.10). Trends toward the higher prevalence of diabetes (28/80, 35.0%, versus 40/160, 25.0%; p = 0.08) and current smoking (26/80, 32.5%, versus 35/160, 21.9%; p = 0.06) were noted among cases, though these did not reach statistical significance. Cardiogenic shock at presentation was slightly higher in cases (6/80, 7.5%, versus 5/160, 3.1%; p = 0.13). Overall, baseline clinical characteristics between cases and controls were largely similar (Table 3).

**Table 3:** Baseline Characteristics (Cases Versus Controls) Significant predictors (p < 0.05) OR, odds ratio; CI, confidence interval; BMI, body mass index; MI, myocardial infarction

Variable	Cases (n = 80)	Controls (n = 160)	P-value	Unadjusted OR (95% CI)
Age (years), mean ± SD	58.0 ± 10.1	57.5 ± 9.8	0.72	1.01 (0.98-1.04)
Male sex, n (%)	62 (77.5)	125 (78.1)	0.92	0.97 (0.51-1.85)
BMI (kg/m²), mean ± SD	27.4 ± 3.7	26.9 ± 3.5	0.31	1.04 (0.96-1.12)
Hypertension, n (%)	42 (52.5)	75 (46.9)	0.38	1.25 (0.75-2.08)
Diabetes mellitus, n (%)	28 (35.0)	40 (25.0)	0.08	1.64 (0.94-2.86)
Current smoking, n (%)	26 (32.5)	35 (21.9)	0.06	1.73 (0.97-3.09)
Dyslipidemia, n (%)	34 (42.5)	58 (36.3)	0.34	1.30 (0.77-2.19)
Prior MI, n (%)	12 (15.0)	18 (11.3)	0.41	1.39 (0.64-3.02)
Cardiogenic shock, n (%)	6 (7.5)	5 (3.1)	0.13	2.50

Men had higher hemoglobin (13.5 ± 1.2 g/dL versus 12.0 ± 1.0 g/dL; p < 0.001) and slightly higher left ventricular ejection fraction (46% versus 42%; p = 0.036) compared to women. Troponin, renal function, platelet count, and random blood glucose were similar. Procedural features, including vascular access, stent type, the number of diseased arteries, pre- and post-dilatation, and infarct-related artery distribution, were comparable between sexes (Tables 4, 5). Thrombus aspiration and repeat PCI were similarly performed in both men and women, indicating equitable management.

**Table 4:** Clinical and Procedural Characteristics of Male and Female Patients With Acute Stent Thrombosis Following Primary PCI Procedural characteristics of early stent thrombosis cases by sex (n = 80). Data are presented as numbers (%). Statistical tests used χ² test or Fisher’s exact test as appropriate DES, drug-eluting stent; BMS, bare-metal stent; PCI, percutaneous coronary intervention; GP IIb/IIIa, glycoprotein IIb/IIIa; OR, odds ratio; CI, confidence interval; χ², chi-square

Variable	Male (n = 62)	Female (n = 18)	Total (n = 80)	Test	P-value	Unadjusted OR (95% CI)
Radial access, n (%)	46 (74.2%)	14 (77.8%)	60 (75.0%)	χ² = 0.09	0.76	0.80 (0.24-2.66)
Femoral access, n (%)	16 (25.8%)	4 (22.2%)	20 (25.0%)	χ² = 0.09	0.76	1.25 (0.38-3.29)
DES use, n (%)	54 (87.1%)	14 (77.8%)	68 (85.0%)	χ² = 1.56	0.21	2.06 (0.57-7.41)
BMS use, n (%)	8 (12.9%)	4 (22.2%)	12 (15.0%)	χ² = 1.56	0.21	0.49 (0.14-1.76)
Stent diameter < 2.75 mm, n (%)	20 (32.3%)	6 (33.3%)	26 (32.5%)	χ² = 0.00	0.96	0.95 (0.31-2.91)
Stent length > 20 mm, n (%)	28 (45.2%)	7 (38.9%)	35 (43.8%)	χ² = 0.15	0.70	1.27 (0.45-3.57)
Pre-dilatation, n (%)	22 (35.5%)	6 (33.3%)	28 (35.0%)	χ² = 0.02	0.88	1.11 (0.38-3.20)
Post-dilatation, n (%)	24 (38.7%)	6 (33.3%)	30 (37.5%)	χ² = 0.14	0.71	1.26 (0.44-3.64)
Thrombus aspiration, n (%)	28 (45.2%)	8 (44.4%)	36 (45.0%)	χ² = 0.00	0.96	1.03 (0.38-2.79)
GP IIb/IIIa inhibitor use, n (%)	22 (35.5%)	6 (33.3%)	28 (35.0%)	χ² = 0.02	0.88	1.11 (0.38-3.20)
Direct stenting, n (%)	30 (48.4%)	8 (44.4%)	38 (47.5%)	χ² = 0.06	0.81	1.18 (0.44-3.15)

**Table 5:** Procedural Characteristics (Cases Versus Controls) Significant predictors (p < 0.05) OR, odds ratio; CI, confidence interval; GP IIb/IIIa, glycoprotein IIb/IIIa; DES, drug-eluting stent; TIMI, thrombolysis in myocardial infarction

Variable	Cases (n = 80)	Controls (n = 160)	P-value	Unadjusted OR (95% CI)
GP IIb/IIIa inhibitor use	28/80 (35.0%)	85/160 (53.1%)	0.01	0.47 (0.28-0.80)
Stent length > 20 mm	35/80 (43.8%)	55/160 (34.4%)	0.12	1.49 (0.89-2.50)
Post-dilatation	30/80 (37.5%)	85/160 (53.1%)	0.02	0.53 (0.31-0.91)
Stent under-expansion	18/80 (22.5%)	20/160 (12.5%)	0.04	2.03 (1.03-4.00)
Radial access	60/80 (75.0%)	125/160 (78.1%)	0.58	0.84 (0.45-1.56)
Pre-dilatation	28/80 (35.0%)	70/160 (43.8%)	0.18	0.69 (0.41-1.17)
Stent diameter < 2.75 mm	26/80 (32.5%)	35/160 (21.9%)	0.06	1.73 (0.97-3.09)
Thrombus aspiration	36/80 (45.0%)	95/160 (59.4%)	0.03	0.56 (0.33-0.94)
DES use	68/80 (85.0%)	145/160 (90.6%)	0.19	0.57 (0.25-1.30)
Pre-procedure TIMI 0 flow	45/80 (56.3%)	75/160 (46.9%)	0.14	1.47 (0.88-2.45)
Final TIMI flow < 3	22/80 (27.5%)	25/160 (15.6%)	0.02	2.03 (1.11-3.71)

Compared with controls, early ST cases were more likely to have final TIMI flow < 3 (22/80, 27.5%, versus 25/160, 15.6%; p = 0.02; OR: 2.03; 95% CI: 1.11-3.71) and stent under-expansion (18/80, 22.5%, versus 20/160, 12.5%; p = 0.04; OR: 2.03; 95% CI: 1.03-4.00). The use of glycoprotein (GP) IIb/IIIa inhibitors (28/80, 35.0%, versus 85/160, 53.1%; p = 0.01; OR: 0.47; 95% CI: 0.28-0.80) and post-dilatation (30/80, 37.5%, versus 85/160, 53.1%; p = 0.02; OR: 0.53; 95% CI: 0.31-0.91) was less frequent among cases, potentially reflecting procedural differences. Other variables such as stent type, radial access, pre-dilatation, and thrombus aspiration were not statistically different (Table 5).

Acute stent thrombosis occurred predominantly within 24 hours of PCI in both sexes. Chest pain was the most common presenting symptom (38/62 men, 61.3%; 12/18 women, 66.7%), followed by ECG changes and hemodynamic instability. Immediate PCI was the first-line therapy in most cases (48/62 men, 77.4%; 14/18 women, 77.8%), while the remainder received medical therapy alone. There were no significant sex differences in timing, clinical presentation, or immediate management (Tables 6, 7).

**Table 6:** Acute Stent Thrombosis and Immediate Management The distribution and management of acute stent thrombosis among male and female patients. The timing of thrombosis is classified as immediate (≤1 hour) and acute (1-24 hours). Only patients meeting the inclusion criteria (acute stent thrombosis occurring during the index hospitalization) were included. χ² values are reported where applicable; for variables with small cell counts, p-values were calculated using Fisher’s exact test PCI, percutaneous coronary intervention; ECG, electrocardiographic; χ², chi-square

Variable	Male (n = 62)	Female (n = 18)	χ²/Fisher’s test	P-value
Timing of thrombosis (≤24 hours), n (%)	Immediate (≤1 hour): 10 (16.1%)	Immediate (≤1 hour): two (11.1%)	0.21	0.65
	Acute (1-24 hours): six (9.7%)	Acute (1-24 hours): two (11.1%)		
Clinical manifestation, n (%)	Chest pain: 38 (61.3%)	Chest pain: 12 (66.7%)	0.44	0.80
	ECG changes: 14 (22.6%)	ECG changes: four (22.2%)		
	Hemodynamic instability: 10 (16.1%)	Hemodynamic instability: two (11.1%)		
Thrombus aspiration used, n (%)	Yes: 28 (45.2%)	Yes: eight (44.4%)	-	0.96
Immediate management strategy, n (%)	Repeat PCI: 48 (77.4%)	Repeat PCI: 14 (77.8%)	-	0.99
	Medical therapy: 14 (22.6%)	Medical therapy: four (22.2%)		

**Table 7:** Timing and Clinical Presentation of AST Data are presented as numbers (%). Statistical test used: chi-square (χ²) test. P-values <0.05 were significant ST, stent thrombosis; PCI, percutaneous coronary intervention; OR. odds ratio; CI, confidence interval; ECG, electrocardiographic; AST, acute ST

Characteristic	Male (n = 62)	Female (n = 18)	Total (n = 80)	χ²	P-value	Unadjusted OR (95% CI)
Acute ST (≤24 hours post-PCI)	16 (25.8%)	4 (22.2%)	20 (25.0%)	0.09	0.76	1.22 (0.36-4.09)
Subacute ST (24 hours to 30 days)	46 (74.2%)	14 (77.8%)	60 (75.0%)	0.09	0.76	0.81 (0.25-2.61)
Chest pain	38 (61.3%)	12 (66.7%)	50 (62.5%)	0.44	0.80	0.81 (0.29-2.25)
ECG changes	14 (22.6%)	4 (22.2%)	18 (22.5%)	0.00	0.98	1.02 (0.28-3.75)
Hemodynamic instability	10 (16.1%)	2 (11.1%)	12 (15.0%)	0.28	0.60	1.53 (0.30-7.76)
Repeat PCI	48 (77.4%)	14 (77.8%)	62 (77.5%)	0.00	0.99	0.97 (0.29-3.27)
Medical therapy alone	14 (22.6%)	4 (22.2%)	18 (22.5%)	0.00	0.99	1.03 (0.31-3.42)

Table 5 describes the timing of stent thrombosis events and their clinical presentation. Acute stent thrombosis occurred predominantly within 24 hours of PCI in both sexes. Chest pain was the most common presenting symptom, followed by ECG changes and hemodynamic instability. There were no significant sex differences in timing or clinical presentation, highlighting that early AST manifests similarly in men and women.

Heart failure was observed in 18/80 patients (14 men, 22.6%; four women, 22.2%), recurrent ischemia in 10/80 (eight men, 12.9%; two women, 11.1%), and arrhythmias in 12/80 (10 men, 16.1%; two women, 11.1%). Other events included acute kidney injury (8/80, 10%), bleeding (6/80, 7.5%), need for mechanical support (4/80, 5%), and in-hospital mortality (6/80, 7.5%). The median coronary care unit (CCU) stay was three days (IQR: 2-5), and total hospital stay was six days (IQR: 4-8), with no significant differences between sexes (Table 8).

**Table 8:** In-Hospital Outcomes Data are presented as numbers (%) or medians (IQR). Statistical tests used: χ² test or Fisher’s exact test for categorical variables and Mann-Whitney U test for continuous non-normally distributed variables CCU, coronary care unit; OR, odds ratio; CI, confidence interval; IQR, interquartile range; χ², chi-square

Outcome	Male (n = 62)	Female (n = 18)	Total (n = 80)	Test	P-value	Unadjusted OR (95% CI)
Recurrent ischemia	8 (12.9%)	2 (11.1%)	10 (12.5%)	Fisher = 0.03	0.86	1.18 (0.24-5.84)
Heart failure	14 (22.6%)	4 (22.2%)	18 (22.5%)	χ² = 0.00	0.97	1.02 (0.28-3.72)
Arrhythmias	10 (16.1%)	2 (11.1%)	12 (15.0%)	χ² = 0.28	0.60	1.53 (0.30-7.76)
Bleeding events	4 (6.5%)	2 (11.1%)	6 (7.5%)	Fisher = 0.49	0.48	0.56 (0.10-3.15)
Acute kidney injury	6 (9.7%)	2 (11.1%)	8 (10.0%)	Fisher = 0.02	0.90	0.86 (0.16-4.56)
Mechanical support	3 (4.8%)	1 (5.6%)	4 (5.0%)	Fisher = 0.02	0.92	0.85 (0.09-7.71)
CCU stay (days), median (IQR)	3 (2-5)	3 (2-5)	3 (2-5)	U = 560	0.52	-
Total hospital stay (days), median (IQR)	6 (4-8)	6 (4-9)	6 (4-8)	U = 570	0.60	-
In-hospital mortality	5 (8.1%)	1 (5.6%)	6 (7.5%)	Fisher = 0.12	0.73	1.50 (0.17-12.8)

## Discussion

The findings of this study, which included 80 patients with early stent thrombosis (≤30 days) following primary PCI, both align with and differ from previously published literature. Large multicenter registries have consistently demonstrated that acute stent thrombosis is an uncommon but clinically significant complication of primary PCI. In a cohort of 5,842 patients with STEMI undergoing primary PCI, definite early stent thrombosis occurred in approximately 1.7% of cases, with an additional 1.8% occurring in the subacute phase (24 hours to 30 days). Although infrequent, these events carry a substantial risk of adverse clinical outcomes [3,9].

In the present study, the overall incidence of stent thrombosis in the broader STEMI population could not be determined; however, the observed patterns of early thrombotic events and associated complications, particularly recurrent chest pain, hemodynamic instability, and early adverse outcomes, are consistent with prior reports. Previous studies have identified several procedural factors, including coronary dissection, stent undersizing, and suboptimal stent deployment, as important predictors of early stent thrombosis. These observations further emphasize the critical role of optimal procedural technique and strict adherence to dual antiplatelet therapy in reducing thrombotic risk [2,3,10-13]. In contrast, our analysis did not demonstrate significant differences in procedural characteristics between male and female patients, and procedural variables did not emerge as strong independent predictors, likely due to sample size limitations or contemporary PCI practice improvements.

An observational prospective study of 1,756 patients undergoing primary PCI reported an overall stent thrombosis incidence of 4.9%, comprising 1.3% acute (≤24 hours) and 3.6% subacute (24 hours to 30 days) events. Male sex, elevated left ventricular end-diastolic pressure, and impaired pre-procedural coronary flow were identified as independent predictors [12]. While our cohort similarly demonstrated male predominance and early thrombotic events, we did not observe statistically significant associations with these predictors, probably due to limited statistical power and low event numbers.

Regarding in-hospital outcomes, prior studies reported high mortality among patients with stent thrombosis after PCI. One study documented in-hospital mortality of 27% in patients with STEMI with stent thrombosis, highlighting the severe prognostic implications despite prompt intervention [13]. In contrast, the in-hospital mortality rate in our cohort was lower (7.5%), though patients experienced a substantial burden of complications, including heart failure and recurrent ischemia. These differences may reflect variations in baseline characteristics, risk profiles, treatment strategies, and supportive care measures.

Evidence consistently suggests that the earlier occurrence of stent thrombosis is associated with poorer short- and long-term outcomes, including higher mortality and recurrent ischemic events [13-15]. Data from the Ottawa Heart Institute STEMI Registry further demonstrated that early stent thrombosis is associated with significantly increased in-hospital and 30-day mortality [15]. Although our study included a matched control group without stent thrombosis, the observed adverse events reinforce the concept that early stent thrombosis remains a condition with serious therapeutic and prognostic implications.

Finally, risk factors frequently reported in the literature, such as diabetes mellitus, hypertension, suboptimal stent deployment, and non-adherence to dual antiplatelet therapy, have been consistently associated with the increased risk of stent thrombosis and poor outcomes [2,16,17]. Although our analysis did not identify statistically significant associations for these factors, likely due to limited power, the findings underscore the importance of optimizing procedural quality and managing modifiable risk factors to reduce stent thrombosis burden.

Strengths and limitations

This retrospective case-control study provides a comprehensive evaluation of clinical characteristics, procedural factors, and in-hospital outcomes associated with early stent thrombosis (≤30 days) following primary PCI. Structured data collection from medical records, catheterization laboratory reports, and electronic hospital databases enhanced completeness and reliability. The inclusion of a matched control group without stent thrombosis allowed a comparative assessment of potential risk factors. Standardized definitions, including those proposed by the Academic Research Consortium (ARC), improved methodological rigor [7,8].

Limitations include a modest sample size of early stent thrombosis cases (n = 80) and limited female representation (n = 18), reducing statistical power for sex-specific analyses. The single-center and retrospective design may limit generalizability and introduce residual confounding despite cross-verification. Long-term outcomes beyond hospital discharge were not assessed, precluding the evaluation of late morbidity and mortality. Some procedural or clinical variables may have lacked power to detect statistically significant associations.

Practical implications and future perspectives

These findings emphasize the importance of the early recognition and prompt management of early stent thrombosis following primary PCI. The optimization of procedural techniques, careful stent deployment, the achievement of adequate post-procedural TIMI flow, and adherence to guideline-directed dual antiplatelet therapy are critical to reducing early thrombotic events. Identifying modifiable procedural factors presents opportunities for quality improvement in interventional practice.

Future research should involve larger, multicenter prospective studies with extended follow-up to better define independent predictors and long-term outcomes of stent thrombosis. The greater representation of women and other underrepresented groups is essential to clarify potential sex-specific differences and improve external validity.

## Conclusions

Patients with early stent thrombosis (≤30 days) following primary PCI generally had similar baseline characteristics to controls, with the exception of smoking, which was more prevalent in certain subgroups. Procedural factors, including stent type, vascular access, stent deployment techniques, and the use of adjunctive medications, were largely standardized across cases. Acute stent thrombosis (≤24 hours post-PCI) occurred predominantly in the early phase and was most commonly managed with repeat PCI. In-hospital outcomes indicated that the majority of patients recovered without major complications; however, a subset experienced adverse events such as heart failure, recurrent ischemia, arrhythmias, acute kidney injury, or bleeding events.

These findings underscore the clinical significance of early and acute stent thrombosis and highlight the importance of prompt recognition, early intervention, and careful post-procedural management to improve patient outcomes. The results also reflect the ongoing challenges of managing this infrequent but serious complication of primary PCI and emphasize the need for preventive strategies, the optimization of procedural techniques, and strict adherence to guideline-recommended dual antiplatelet therapy. Future studies should aim for larger, multicenter cohorts with long-term follow-up, particularly focusing on high-risk groups and improving female representation, to better delineate risk factors and refine management strategies.
